# Supplementary material for: Experimental study on repair of fractured rock mass by microbial induction technology
Source: R Soc Open Sci. 2019 Nov 20;6(11):191318. doi: 10.1098/rsos.191318 (PMC6894604; doi:10.1098/rsos.191318)
Supplement: Figure illustrations during the experiment [file rsos191318supp1.docx]

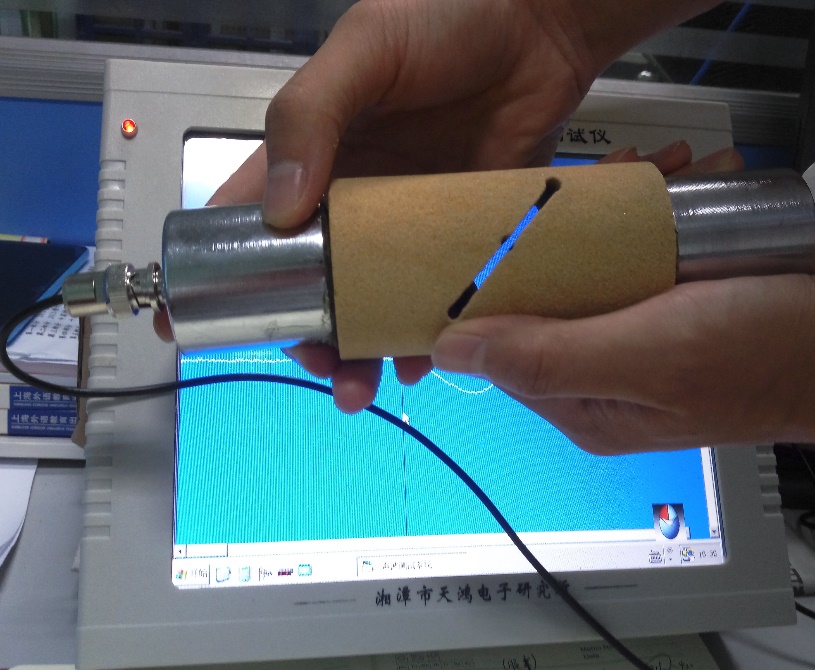


Figure 1 Using the rock acoustic wave parameter tester to obtain the longitudinal wave velocity


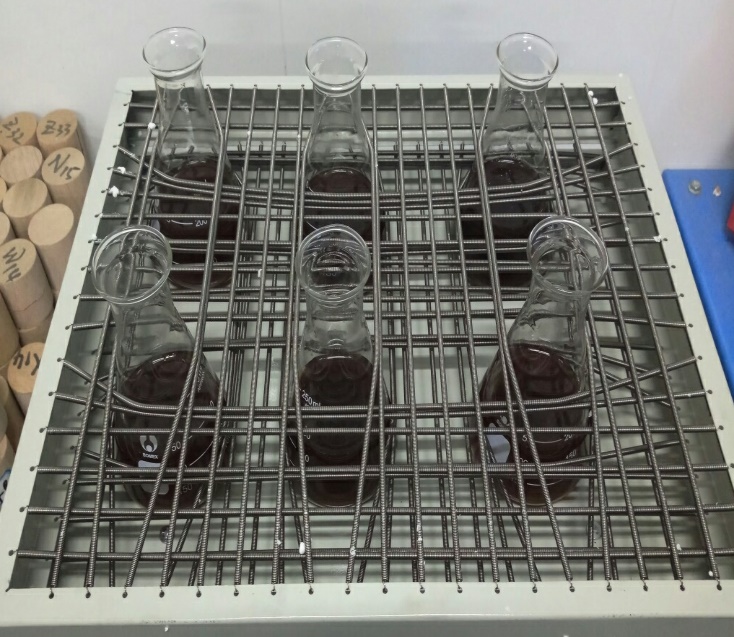


Figure 2 High concentration of bacteria in a constant temperature shaker


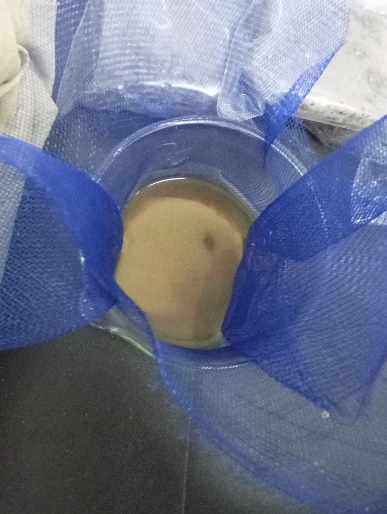

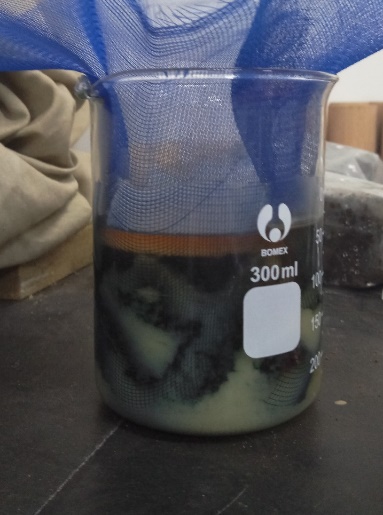


Figure 3 Microbial repair test process


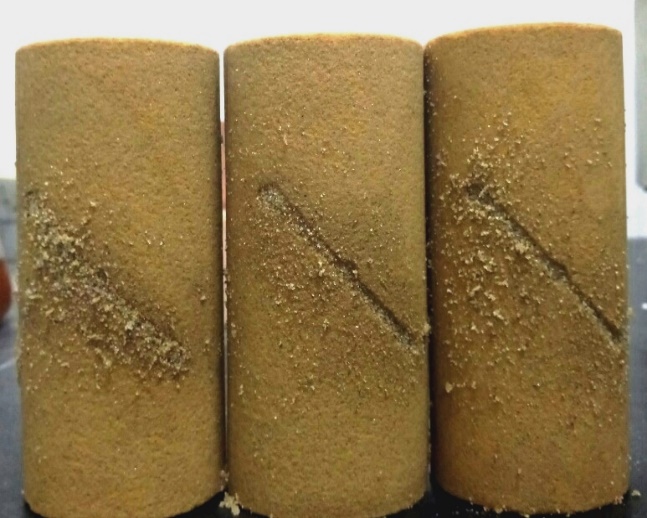


Figure 4: Sand filled in prefabricated cracks


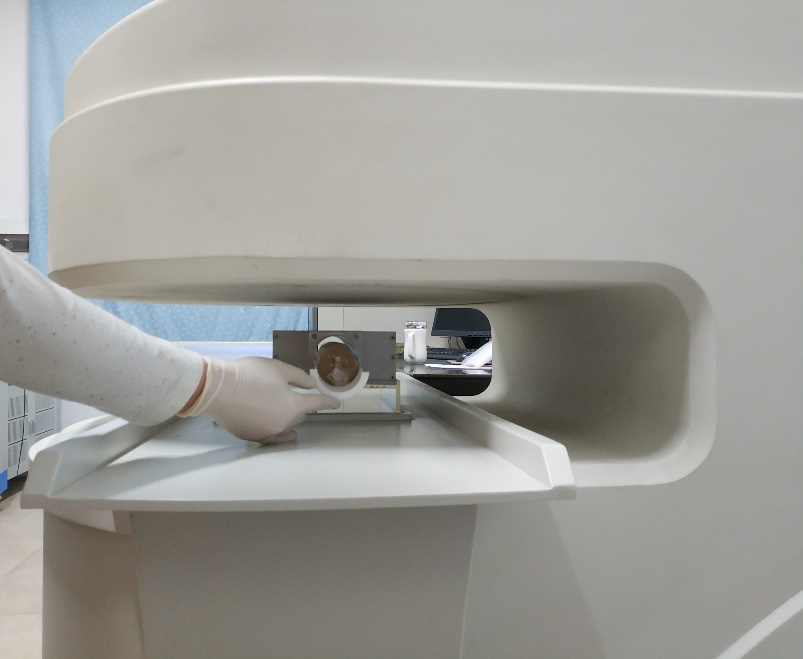


Figure 5 Place the sample in a magnet box for NMR testing
